# Effect of Superabsorbent Polymer on the Properties of Concrete

**DOI:** 10.3390/polym9120672

**Published:** 2017-12-04

**Authors:** Juntao Dang, Jun Zhao, Zhaohua Du

**Affiliations:** 1 College of Civil Engineering, Zhengzhou University, Zhengzhou 450001, China; dt_wolf@163.com; 2 College of Mechanics and Engineering Science, Zhengzhou University, Zhengzhou 450001, China; 3 Multi-Functional Design and Research Academy, Zhengzhou University, Zhengzhou 450052, China; hndzh1972@163.com

**Keywords:** concrete, superabsorbent polymer, internal curing, workability, strength, shrinkage, durability

## Abstract

Incorporating superabsorbent polymer (SAP), which has the abilities of absorption and desorption in concrete can achieve the effect of internal curing. The influences of the volume, particle size and ways of entrained water of SAP on the workability, compressive strength, shrinkage, carbonation resistance and chloride penetration resistance of concrete were analyzed through the macroscopic and microscopic test. The results show that pre-absorbed SAP can increase the slump of the mixture, but SAP without water absorption and pre-absorbed SAP with the deduction of internal curing water from mixing water can reduce the slump. The improvement effects of SAP on compressive strength of concrete increase gradually with the increase of age. Especially from 28 days, the compressive strength of concrete increases obviously. At later age, the compressive strengths of SAP concrete under natural curing environment exceed the strength of reference concrete under natural curing environment and nearly reach the strengths of reference concrete under standard curing environment. SAP effectively reduces the shrinkage of concrete, improves the concrete’s abilities of carbonation resistance and chloride penetration resistance. The microscopic test results show that SAP can effectively improve the micro structure and make the pore structure refined. When SAP is added into concrete, the gel pores and small capillary pores are increased, the size of big capillary pores and air pores are reduced.

## 1. Introduction

Concrete is a kind of porous, multiphase at all scales, and heterogeneous complex system [1]. The degree of hydration of cement plays a decisive role in the strength and compactness of concrete. Optimizing the pore structure, reducing porosity and reducing macro pores are the necessary improvement measures for high strength and high durability concrete [2]. The research on hydration mechanism of cement showed that the inner structure of concrete had fine pore size and compact structure when the water binder ratio was relatively low [3,4]. However, there will be more unhydrated cement and mineral admixture particles, which will result in the decrease of internal relative humidity and the increase of the autogenous shrinkage of concrete [5,6]. Water curing is a very important technical measure for concrete in order to ensure the hydration process of cementitious materials in concrete. Effective water curing measures should ensure the enough moisture environments inside and outside the concrete which are necessary for hydration. If the moisture is not enough, it will cause early shrinkage and even the cracking of concrete, which will adversely affect the growth of strength and durability of concrete at later age. In practical engineering, manual operation and external curing are usually adopted, which is very difficult to produce timely and adequate curing. 

The internal curing method has been studied for some years which may be an effective way to ensure the hydration process in concrete. Water is introduced into the concrete in advance by absorbent material like superabsorbent polymer (SAP), so that some of the water acts as internal curing water from the inside out, in order to achieve the effect of internal curing in the hydration process of the cementitious material. When the relative humidity inside the cement matrix gradually drops, the absorbent material can slowly release water to supplement the water consumption of cement hydration. The hydration process continued to generate hydration products to fill the pores of cement paste, reduce the micro cracks in cement matrix, and relieve the autogenous shrinkage and drying shrinkage. Then the strength and durability of the concrete can be improved [7,8,9].

SAP is a common water absorbent material with excellent water absorption and desorption capacity. In recent years, relevant research work on SAP concrete has been carried out internationally, and the influence of SAP on the construction performance, strength and durability of concrete has been studied [10,11]. It has been shown that the addition of SAP has both positive and negative effects on concrete properties. The research conducted by Dudziak and Mechtcherine [12] indicated that because the SAP in concrete absorbed moisture in the mixture and reduces the slump of the concrete, additional internal curing water absorbed by the SAP was needed to compensate for the moisture in concrete. Snoeck and Schaubroeck [13] compared the performance of concrete with different kinds of SAP. The results showed that if the particle size was small, the water absorption rate and the volume of SAP had a negative impact on the performance of concrete. When internal curing water was absorbed by SAP, the reduction of workability could be compensated to a certain extent [12,14]. However, SAP will leave pores when water is released. Adding SAP which absorbed additional internal curing water increases porosity, and this may have a negative effect on the strength of concrete [15,16,17]. Studies [4,18,19,20,21] have also shown that although the swelling of SAP affects the development of early pore structures, the water absorbed by the SAP will gradually release when the relative humidity of the concrete decreases. It will effectively alleviate the autogenous shrinkage and promote hydration of cementitious materials. Therefore, the strength of concrete may be enhanced. Although SAP can effectively alleviate autogenous shrinkage, there is a lack of experimental studies on the combined effects of autogenous shrinkage and drying shrinkage in the natural environment at long ages. 

The incorporation of SAP increases the porosity of concrete which may affect the durability of concrete. However, SAP with the absorption and desorption properties can change the water distribution in concrete. So that the process of cement hydration is promoted, the microstructure of cement matrix is improved, and the adverse effects of voids released by SAP can be offset. Hasholt and Jensen [22] concluded that the large volume of gel solid made the pore system become more tortuous and the porosity become less. Thus the chloride ion diffusion coefficient of concrete was reduced. 

In this paper, the influence of the volume, particle size and ways of entrained water of SAP on the workability, compressive strength, shrinkage, carbonation resistance and chloride penetration resistance of concrete have been experimentally studied. Based on the microscopic test results of mineral compositions, pore structures and morphology of hydration products, the mechanism of SAP on the performance of concrete has been studied.

## 2. Experimental Investigations

### 2.1. Materials

**Figure 1:**
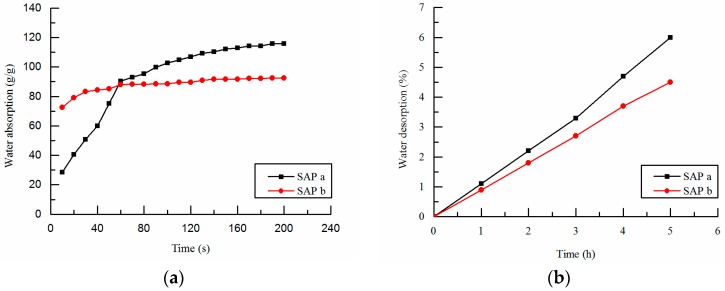
Time dependent water absorption and desorption behavior of superabsorbent polymer (SAP): (**a**) water absorption; (**b**) water desorption.

An ordinary Portland cement (Tianrui group cement Co. Ltd., Zhengzhou, China) was used. The physical and chemical compositions of the cement are presented in Table 1 and Table 2. Chemical composition of fly ash used as mineral admixture is presented in Table 2. The coarse aggregate used was crushed granite of size ranging from 5–20 mm. The fine aggregate was natural river sand with a fineness modulus of 2.37. Tap water was used as mixing water and internal curing water. Polycarboxylate superplasticizer (YJK-01 type, solid content 30% and water reducing rate 27%, Henan zhenxiang road & bridge science and technology Co. Ltd., Zhengzhou, China) was used to guarantee the workability of the reference concrete with a controlled slump of 190 ± 20 mm. The properties of superabsorbent polymer (SAP, Hebei xiguang chemical technology Co. Ltd., Shijiazhuang, China) used as internal curing materials are shown in Table 3. The appearance of SAP is spherical and white powder, swelling and gel state after mixing with water. Under the environment of (20 ± 3) °C, (40 ± 5)%RH, SAP has absorption and desorption capacity measured by the tea bag method. The water absorption and desorption behaviors of SAP are shown in Figure 1. Results indicate that SAP have high water absorption capacity and fast water absorption rate. However, the water release rate of SAP is relatively slow, and the water retention property is good.

### 2.2. Mix Proportion and Specimen Preparation

According to the specification for mix proportion design of ordinary concrete (JGJ55-2011) [23], the specimens were designed and constructed, the influences of the SAP volume, particle size and ways of entrained water on workability, compressive strength, shrinkage and durability of concrete have been analyzed. Mix proportions are shown in Table 4. The ratio of water to binding material (w/b) of reference concrete mixture (PC) was fixed to 0.38. S01, S02 and S03 mean the volumes of SAP are 0.1%, 0.2% and 0.3% of cementitious materials, respectively. The particle sizes of SAP-a are from 250 to 425 μm and the particle sizes of SAP-b are from 150 to 180 μm. The ways of entrained water of SAP are 0 (SAP without water absorption), 10 (SAP pre-absorbed internal curing water) and 10K (pre-absorbed SAP with the deduction of internal curing water from mixing water). SAP pre-absorbed internal curing water was 10 times the weight of dried SAP. 

Mixing program of materials was prepared as follows: (a) the raw materials which is taking as dry state should be weighted accurately. SAP was divided into non-absorbed and pre-absorbed of 10 times the weight of SAP, respectively; (b) The fine aggregate, coarse aggregate, cement and fly ash were poured into the wet mixing bucket and mixed 30 s to ensure the uniformity; (c) Water reducing agent and concrete mixing water were added to the mixture and mixed for 100 s; (d) Non-absorbed SAP or pre-absorbed SAP were added to the mixture and mixed for 60 s to ensure that SAPs were distributed homogenously in the mixture.

All specimens were casted and kept indoors for 24 h, and then demoulded and numbered. The natural curing and standard curing methods were used for reference concrete specimens, which represent PC-Z and PC-B respectively. Only natural curing method was used for internal curing concrete specimens, which represent S. The natural curing concrete specimens were placed in the outdoor environment. The standard curing concrete specimens were stored in the standard curing room where the temperature was (20 ± 2) °C and relative humidity was above 95%.

### 2.3. Testing Methods

#### 2.3.1. Workability

The workability test was carried out according to the standard for test method of performance on ordinary fresh concrete (GB/T50080-2016) [24], and the slumps of the mixtures were measured.

#### 2.3.2. Compressive Strength

The compressive strength test was carried out according to the standard for test method of mechanical properties on ordinary concrete (GB/T50081-2002) [25]. A total of 3 cubes with the size of 150 mm × 150 mm × 150 mm were tested for each batch. The curing environment was divided into standard condition and natural condition. Finally the compressive strength test was performed at 7, 14, 28, 56, 90 and 120 days.

#### 2.3.3. Shrinkage

The shrinkage test was carried out by non-contact method according to the standard for test method of long-term performance and durability of ordinary concrete (GB/T50082-2009) [26]. A total of 3 prismatic specimens with the size of 100 mm × 100 mm × 515 mm were tested for each batch. After casting, the undemoulded specimen was placed in the natural environment immediately for a period of 56 days and the shrinkage data were collected. The testing instrument is non-contact concrete shrinkage deformation tester.

#### 2.3.4. Carbonation Resistance

The carbonation resistance test was carried out according to the standard for test method of long-term performance and durability of ordinary concrete (GB/T50082-2009) [26]. A total of 3 prismatic specimens with the size of 100 mm × 100 mm × 400 mm were tested for each batch. The specimens were cured for 28 days under the corresponding curing conditions. Before the carbonation resistance test, the specimens were placed at 60 °C for drying 48 h. The top and the bottom surfaces of the specimen obtained from prism were painted with paraffin so that only lateral diffusion of CO_2_ could be achieved. The specimens were positioned in an accelerated carbonation chamber which was set at (20 ± 2) °C, (70 ± 5)% of relative humidity and (20 ± 3)% of CO_2_. The carbonation depths were measured at 3, 7, 14 and 28 days using a phenolphthalein solution on the exposed surfaces of split specimens.

#### 2.3.5. Chloride Penetration Resistance

The chloride penetration resistance test was carried out by rapid chloride ions migration coefficient method (RCM) according to the standard for test method of long-term performance and durability of ordinary concrete (GB/T50082-2009) [26]. A total of 3 cubes with the size of 150 mm × 150 mm × 150 mm were tested for each batch. 7 days before chloride penetration resistance test, the cylindrical specimens (Ф100 ± 1 mm, H = 50 ± 2 mm) were drilled from the above cubic specimens. Then the cylindrical specimens were cured under corresponding curing conditions until 28 days. The cylindrical specimens were placed in a chamber with vacuum. Under vacuum, the specimens were immersed in Ca(OH)_2_ solution. Subsequently, the specimens were placed inside rubber cell. The top surface of the specimens was filled with a 0.3 mol/L NaOH anodic solution and the bottom surface of the specimens was filled with a 10% NaCl cathodic solution. Rapid chloride transport (RCM) tests were then performed. After the test, the specimen was taken out to flash the surface, and then cut along the direction of the axis parallel to the specimen. The fracture surface was sprayed with silver nitrate (0.1 mol/L AgNO_3_) solution. Finally the depth of chloride penetration after 15 min was measured and the chloride migration coefficient was computed.

#### 2.3.6. Investigation of the Microstructure

The mercury intrusion porosimetry (MIP) test, X-ray diffraction (XRD) test and scanning electron microscope (SEM) test were carried out to research the microstructural characteristics of concrete. After being cured 56 days, the micro-specimens were taken from the center of samples and placed into anhydrous alcohol to stop cement hydration. The micro-specimens were placed for 24 h in an oven at 60 °C. Then the microscopic experiments were carried out. 

Mercury intrusion porosimetry test was carried out by using AutoPore IV 9500 automatic mercury intrusion apparatus (Micromeritics Instrument Corporation, Shanghai, China) and the measured pore diameters was 0.003–1000 µm. For X-ray diffraction test, the micro-specimens were ground into powder in an agate bowl until the particle size was below 0.08 mm. After vacuum drying, the X ray diffraction test was carried out. The tests were performed over a Braggs angle (2θ) range of 5–60°. The fracture surfaces of the micro-specimens were sputtered a layer of gold. The micro-specimens were observed by scanning electron microscope at different degrees of magnification.

## 3. Results and Discussion

### 3.1. Workability

**Figure 2:**
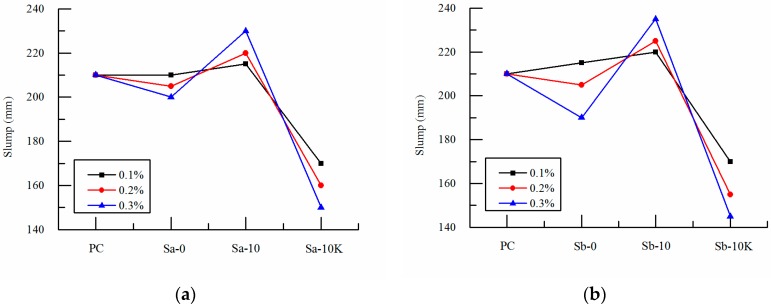
Effects of SAP on slump of concrete: (**a**) SAP-a; (**b**) SAP-b, S-10 and S-10K are pre-absorbed, and S-0 is non-absorbed.

The influence curves of different ways of entrained water and volume on concrete slump for two particle sizes of SAP is shown in Figure 2. As can be seen from Figure 2, the slump of mixture is influenced greatly by the ways of entrained water and the volume of SAP, and is less affected by the change of particle size of SAP when SAP of different ways of entrained water was added in the reference concrete mixture. The slump of mixture varies slightly near the slump of reference concrete with the change of SAP particle size and volume. And the range of change is small when SAP was added directly. But the slump increases when the pre-absorbed SAP was added and the slump increases with the increase of SAP volume. It indicates that the pre-absorbed SAP presents as spherical fine particles and this can play a lubrication role in concrete mixture to reduce the friction between slurry and aggregate. It can also increase the fluidity of the mixture as well as increase the slump of the mixture. The more the amount of SAP is added, the more obvious the effect is. In addition, adding pre-absorbed SAP with the deduction of internal curing water from mixing water has a great influence on the slump. And with the increase of SAP volume, the slump decreases obviously. When the volume of SAP is 0.3%, the slump is reduced to 145 mm. The slump of concrete is about 31% lower than that of the reference concrete. Under the influence of the different ways of entrained water and volume of SAP, the available water for mixing is reduced, thus giving rise to the decrease of fluidity of the matrix slurry.

### 3.2. Compressive Strength

**Figure 3:**
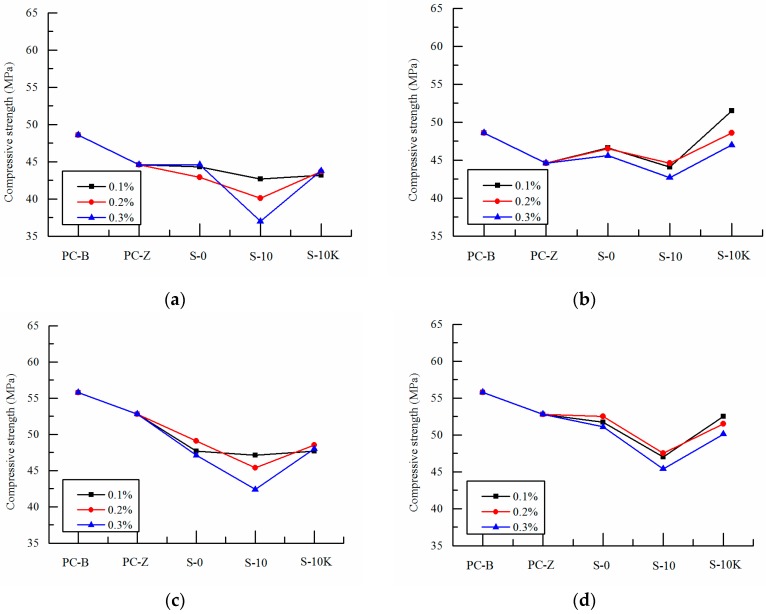
Effects of SAP on compressive strength of concrete at early ages: (**a**) 7 days SAP-a; (**b**) 7 days SAP-b; (**c**) 14 days SAP-a; (**d**) 14 days SAP-b, S-10 and S-10K are pre-absorbed, and S-0 is non-absorbed.

**Figure 4:**
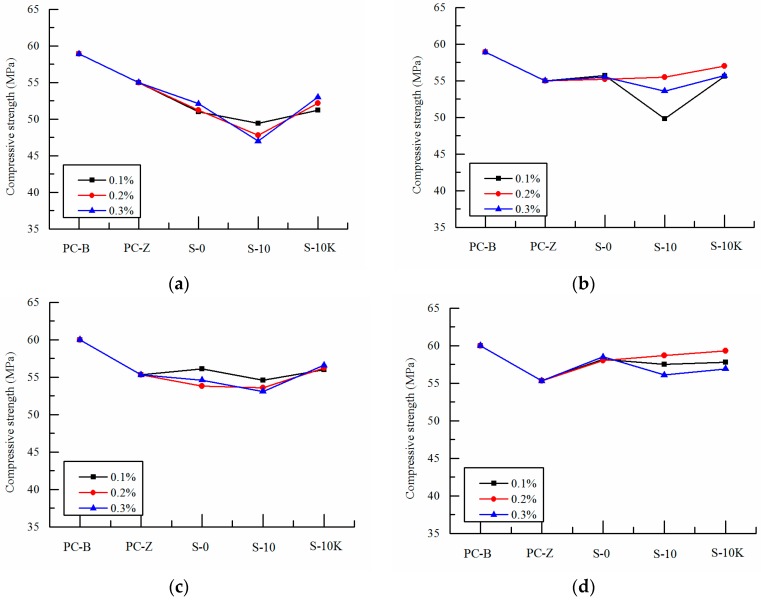
Effects of SAP on compressive strength of concrete at medium ages: (**a**) 28 days SAP-a; (**b**) 28 days SAP-b; (**c**) 56 days SAP-a; (**d**) 56 days SAP-b, S-10 and S-10K are pre-absorbed, and S-0 is non-absorbed.

**Figure 5:**
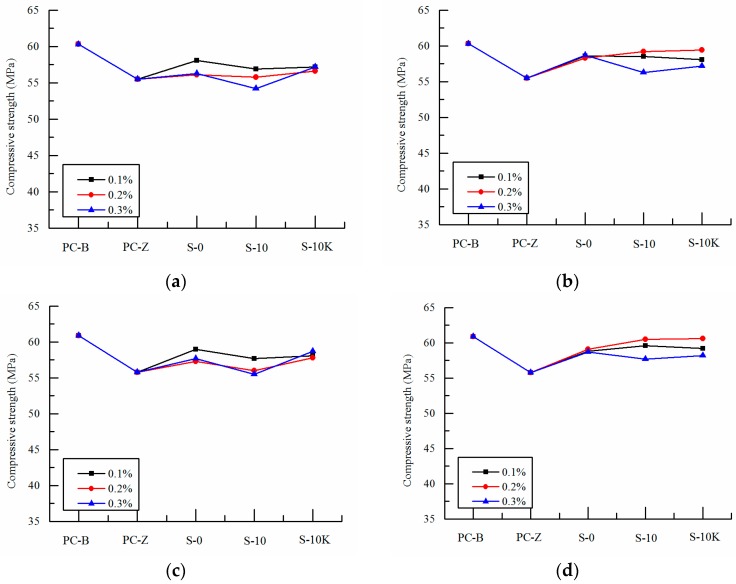
Effects of SAP on compressive strength of concrete at later ages: (**a**) 90 days SAP-a; (**b**) 90 days SAP-b; (**c**) 120 days SAP-a; (**d**) 120 days SAP-b, S-10 and S-10K are pre-absorbed, and S-0 is non-absorbed.

Figure 3, Figure 4 and Figure 5 show the changes of compressive strength of SAP concrete mixed with different particle size, volume and ways of entrained water at early ages (7 days, 14 days), medium ages (28 days, 56 days) and later ages (90 days, 120 days).

As can be seen from Figure 3, with the exception of more than 0.2% amount of pre-absorbed SAP-a, the addition of SAP has little effect on the compressive strength of concrete at 7 days. With the rapid development of early hydration process of cement, SAP has a negative influence on the compressive strength of concrete at 14 days. It shows that the curing function of SAP has not played a leading role at early age and it also has a negative effect on the compressive strength of concrete. On the one hand, this is because in the early development of concrete strength, the pre-absorbed SAP itself or the absorption of free water in concrete result in the expansion in size and the formation of more voids, thus reducing the compressive strength of concrete. On the other hand, the addition of SAP which is soft microcapsules may decrease the compressive strength of concrete [27,28]. And the compressive strengths of curing concrete at early age are greatly affected by the ways of entrained water and the particle size of SAP, and are less affected by the change of SAP volume. With the increasing of SAP volume, larger particle size and pre-absorbed SAP-a cause the additional internal curing water increase gradually. The effective water cement ratio of concrete is increased and the compressive strength decreases linearly. And the compressive strength decreased by 24% at most.

With the increase of ages, the differences of compressive strengths between SAP concrete and the reference concrete decrease gradually which is shown in Figure 4. At 28 days, the compressive strength of SAP-b concrete is basically the same as that of PC-Z reference concrete, which shows that SAP has gradually played the role of internal curing [29,30,31]. However, the strength of SAP-a concrete is still low, and it is affected by its larger particle size or the performance of water absorption and desorption. At the age of 56 days, the strength of the reference concrete no longer increase, but the strength of the internal curing concrete added with SAP continues to grow. The strength of SAP-a concrete increases gradually, which is consistent with the compressive strength of PC-Z reference concrete. While the compressive strength of SAP-b concrete is slightly higher than that of PC-Z reference concrete, the maximum increase is 7.2%. It shows that the internal curing function of SAP has played an important role in concrete. This is because cement hydration reaction has been basically completed at medium age, and the pozzolanic effect of the fly ash in concrete begins. Although the internal humidity of the reference concrete has been unable to meet the second hydration reaction, the internal curing concrete with SAP, which is pre-absorbed or absorbs free water from concrete, can effectively provide the water needed for the second hydration reaction of cement with fly ash paste in the long time. More hydration products are produced and pore structure is refined. Other scholars have shown that the internal curing effect can effectively promote the second hydration reaction of the long age as well as offset the adverse effects caused by SAP’s own void, thus improving the compressive strength of concrete [32,33,34].

As can be seen from Figure 5, the compressive strength of the SAP curing concretes at 90 days and 120 days are basically higher than that of PC-Z reference concrete, which indicates that the internal curing effect of SAP has been effectively worked. This is because the pozzolanic activity of fly ash is nearly fully excited with the development of age. Although the early water absorption of SAP results in larger voids, which exerts negative effect on strength, the internal curing water is better stored and is beneficial to release water in the later stage of SAP to meet the second hydration reaction when pre-absorbed SAP is added in concrete. And the compressive strength is further improved effectively. At the age of 120 days, the compressive strength of 0.2% amount of pre-absorbed SAP-b is basically the same with that of the pre-absorbed SAP with the deduction of internal curing water from mixing water. And the compressive strength even reaches to that of PC-B reference concrete which under the standard curing environment. It shows that the incorporation of pre-absorbed SAP changed the way of water storage which is beneficial to the water storage in concrete. Especially when the water in concrete is lost in later age, the pre-absorbed SAP-b store sufficient moisture which is beneficial to the internal curing effect of SAP. 

In the later age, the compressive strength of the internal curing concrete is greatly affected by the particle size of SAP. The strength of SAP-b concrete is generally higher than that of SAP-a concrete, which shows that SAP-b is more suitable than SAP-a in playing the role of internal curing. This is because the change of the particle size of SAP has very much to do with the water absorption and desorption properties (Table 3 and Figure 1), and the distribution of SAP in cement matrix as well as the pore size after water release. Thus the extent and scope of internal curing effect of SAP has been changed. The influence of SAP on the mechanical properties is mainly determined by the type of SAP and the dynamics of water absorption and desorption [35]. It is not easy for SAP to well-scattered in concrete and liable to get together causing uneven water absorption when the particle size is small and the water absorption rate is faster. However, the time of water absorption and desorption plays a control role when the particles are large, which will affect the efficiency of SAP water absorption and desorption. Therefore, SAP should have an optimum particle size range to achieve the best water absorption and desorption effect in concrete.

### 3.3. Total Shrinkage

**Figure 6:**
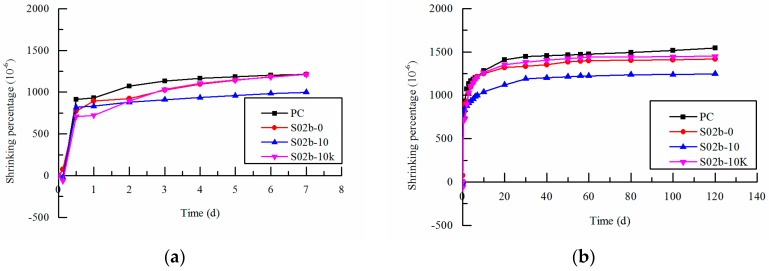
Effects of SAP on total shrinkage of concrete: (**a**) the early age; (**b**) the later age, S02b-10 and S02b-10K are pre-absorbed, and S02b-0 is non-absorbed.

Figure 6 reflects the influence curve of SAP-b variation with different ways of entrained water on total shrinkage in early and later age. As can be seen from Figure 6, the total shrinkage of concrete increases gradually as age goes by. Volume expansion appears at the early age, and then followed by a rapid total shrinkage. However, the shrinkage tends to slow down in later age. Due to the water consumption of cement matrix at early age, the capillarity pressure inside the cement matrix is generated. Thus makes the autogenous shrinkage plays a dominant role [5]. The total shrinkage value of concrete with 0.2% SAP-b content is decreased when compared with the reference concrete at 3 days. The concrete shrinkage values of S-0, S-10 and S-10K decrease by 9%, 20% and 9% respectively in different ways of entrained water. At 7 day age, the shrinkage value of S-0 and S-10K are consistent with that of the reference concrete. But the shrinkage value of the pre-absorbent SAP concrete is 18% lower than that of the reference concrete. The drying shrinkage, which is caused by the uneven scattered internal water in concrete and the evaporation of surface water, is the major factor in shrinkage of concrete [35,36]. The concrete shrinkage values of S-0, S-10 and S-10K decrease by 5%, 17% and 2% respectively in different ways of entrained water compared with the reference concrete at 56 days. As can be seen from the microscopic test, SAP can effectively supply the water needed for the second hydration reaction of cement fly ash system. So that the pore structure tends to be compact, the drying water loss rate slows down and the shrinkage rate decreases. At 120 days, it can be seen from the comparison that the concrete shrinkage values of S-0, S-10 and S-10K decrease by 8%, 19% and 6% respectively in different ways of entrained water which shows that SAP plays an internal curing role. This is because at the early age of concrete hardening, SAP adds water to the capillary pores of cement matrix which can maintain the water in capillary pores and reduce the stress of capillary as well as relieve the autogenous shrinkage of concrete. When the relative humidity inside the concrete decreases at later age, SAP can release the internal water stored to supply for the water consumption in concrete. It also can reduce the evaporation rate of external water as well as alleviate the relative humidity gradient of concrete so as to improve the drying shrinkage of concrete.

Compared with the reference concrete, the shrinkage value is slightly decreased when non-absorbed SAP or pre-absorbed SAP with the deduction of internal curing water from mixing water is added. However, the total shrinkage of pre-absorbed SAP is reduced remarkably. It indicates that pre-absorbed SAP can better improve the shrinkage of concrete. This is because with the change of ways of entrained internal curing water, the storage form of SAP changed. Especially the pre-absorbed SAP can effectively store additional water and improve the effectiveness of internal maintenance. In the process of concrete hardening, SAP release water to the capillary pore of cement matrix in order to extend the period of saturation as well as alleviate the shrinkage of concrete. The added internal curing water and the absorption of free water from the mixture have different effects on the internal humidity of the concrete, which results in different degrees of shrinkage relieving.

### 3.4. Carbonation Resistance

**Figure 7:**
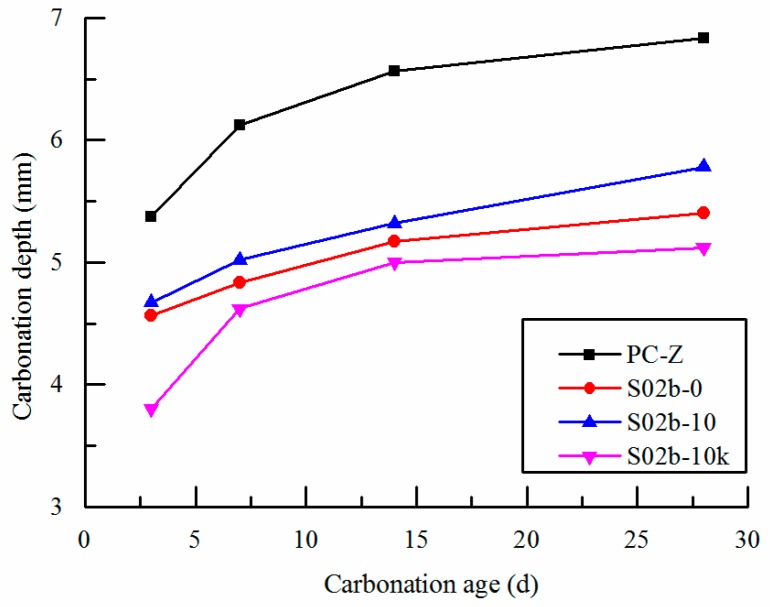
Effects of SAP on carbonation depth of concrete. S02b-10 and S02b-10K are pre-absorbed, and S02b-0 is non-absorbed.

Figure 7 shows the influence curve of SAP-b on the carbonation depth with different ways of entrained water at 28 days. As can be seen from Figure 7, with the growth of the carbonation time, the carbonation depth increases constantly. Compared with the reference concrete, the carbonation depth of concrete with 0.2% SAP-b is reduced. It shows that SAP concrete has better carbonation resistance. The carbonation resistance of concrete is related to the compactness of the matrix. And the concrete carbonation depth of S-0, S-10 and S-10K decrease by 25%, 21% and 15% respectively in different ways of entrained water. This shows that the depth of carbonation decreases with the decrease of effective water binder ratio. Moreover, the way of entrained water of SAP makes the effective water binder ratio change, thus affecting the carbonation performance of concrete. When adding SAP directly or adding pre-absorbed SAP with the deduction of internal curing water from mixing water, the effective water binder ratio is reduced because of the water absorption property. So that it is beneficial to form a compact cement matrix. The reason is because the SAP absorbs free water inside concrete or absorbs internal curing water in advance. In the process of concrete hardening, the water of SAP is released to the surrounding environment, which promotes the further hydration of cement matrix, thus increasing the compactness of concrete.

### 3.5. Chloride Penetration Resistance

**Figure 8:**
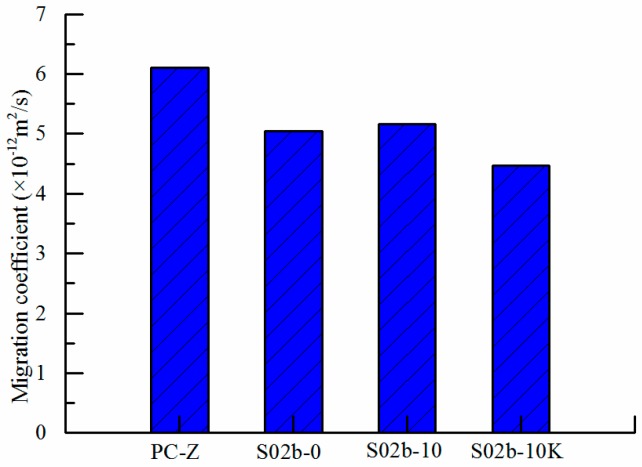
Effects of SAP-b on chloride ion migration coefficient of concrete. S02b-10 and S02b-10K are pre-absorbed, and S02b-0 is non-absorbed.

Figure 8 shows the influence curve of SAP-b on the diffusion coefficient of chloride ion at 28 days. The variation of chloride ion diffusion coefficient is similar to that of carbonation depth with different ways of entrained water of SAP. As can be seen from Figure 8, the incorporation of SAP into concrete has a great influence on the chloride ion diffusion coefficient of concrete. Compared with the PC-Z reference concrete, the chloride ion diffusion coefficient of SAP-b concrete with 0.2% volume SAP decreased, which indicates that the internal curing concrete mixed with SAP remarkably improved the resistance to chloride ion corrosion of concrete. This is because of the incorporation of SAP improves cement hydration environment, and promotes the second hydration reaction between hydration products in cement and active components in fly ash. More hydration products are produced to make the concrete more compact. The pore structure tends to become fine, and the connection among the capillary pores is cut off, which effectively limits the diffusion of Cl^−^ and enhances the resistance to chloride ion penetration.

At the same time, the chloride ion diffusion coefficient is greatly affected by the ways of entrained water of SAP. And the Chloride ion diffusion coefficients of S-10K, S-0 and S-10 decrease by 27%, 17% and 15% respectively in different ways of entrained water. This shows that the diffusion coefficients of chloride ion decrease with the decrease of the effective water binder ratio. In addition, the SAP without water absorption and the pre-absorbed SAP with the deduction of internal curing water from mixing water can reduce the total pore volume and porosity of concrete, so that the compactness of concrete is improved. Other scholars [4,13,37] found that early shrinkage crack is reduced when SAP added. The blocking effect of SAP coated with organic film which is formed by the releasing of SAP leads to poor pore connectivity, thus reducing the diffusion coefficient of chloride ion.

### 3.6. Investigation of the Microstructure

#### 3.6.1. Mercury Intrusion Porosimetry (MIP)

Table 5 reflects the pore structure parameters and distribution of SAP concrete at 56d. Compared with the PC-Z reference concrete, with the exception of the pre-absorbed SAP concrete (S-10), the total pore volume and porosity of internal curing concrete decrease when SAP is added with a proper volume and particle size. The porosity and total pore volume of S-10K and S-0 decrease by 9% respectively in different ways of entrained water. While the porosity and total pore volume of S-10 increase by 2% and 4% respectively due to the different ways of entrained water of SAP. The addition of pre-absorbed SAP increases the effective water binder ratio of concrete, which results in the increase of porosity and total pore volume. The addition of non-absorbed SAP and pre-absorbed SAP with the deduction of internal curing water from mixing water makes the water binder ratio decreased. However, the distribution of pore size of internal curing concrete presents as increased gel pores, small capillary pores, decreased large capillary pores and air pores. It is shown that the pore structure of concrete tends to be refined when SAP is added. This is because the incorporation of SAP can improve the water distribution in the concrete and optimize the hydration process of the cementitious material. Especially during the middle and later ages of the development of cement matrix, it is more favorable for SAP to release water, to promote the pozzolanic reaction of fly ash as well as to produce dense calcium silicate hydrate to reduce the number of communicating pores leading to the outside. This can explain the reasons for the increase of compressive strength and durability. The studies conducted by Lura [37] through X-ray tomography showed that SAP reduced smaller capillary pores. This is because, on the one hand, the hydration reaction is facilitated by the internal curing effect, and the hydration product fills the existing pores. On the other hand, the initial micro-cracks of the cement mortar are reduced and the autogenous shrinkage of the cement matrix is relieved.

#### 3.6.2. X-ray Diffraction (XRD)

The quantitative analysis of XRD test on the component of SAP concrete at 56 days age is shown in Table 6. Quantitative analysis of Ca(OH)_2_ and ettringite in cement-based materials is carried out by the XRD-Rietveld full spectrum fitting method. Compared with the PC-Z reference concrete, the content of Ca(OH)_2_ and ettringite in the internal curing concrete is generally reduced by more than 40% by adding proper amount of SAP. It shows that the incorporation of SAP provides fly ash and Ca(OH)_2_ with water that needed for second hydration reaction. This effectively promotes the formation of hydration products and makes the Ca(OH)_2_ of the interface area of aggregate and mortar reduced greatly. A large amount of stable hydrated calcium silicate is generated, which is filled in the micro pores of the cement mortar, so that the Ca(OH)_2_ is surrounded by the impermeable C-S-H gel, and the quantity and the size of the Ca(OH)_2_ crystal are reduced. It also reduces the formation of expansive salts, which is called ettringite. The reduction of the number and size of Ca(OH)_2_ crystals and ettringite enhances the strength of cement paste, improves the microstructure of matrix, the strength and compactness of concrete. The SEM image test shows that it is difficult to find Ca(OH)_2_ crystal in the cement matrix mixed with SAP, which further confirms the fact that the content of Ca(OH)_2_ decreases in XRD test.

#### 3.6.3. Scanning Electron Microscope (SEM)

**Figure 9:**
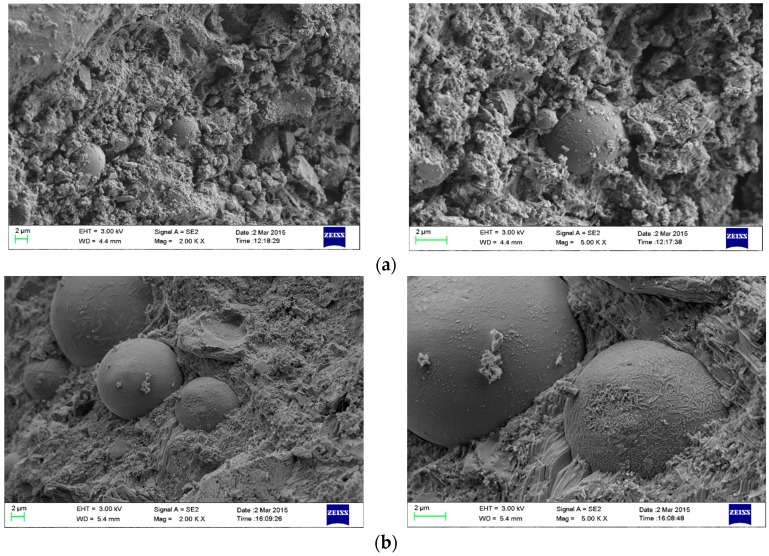

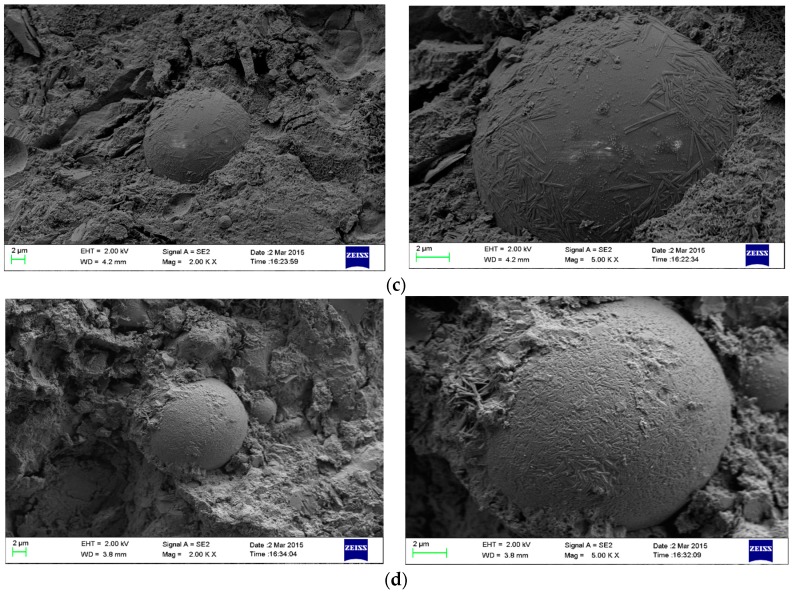
SEM image of cement paste with addition of SAP at 56 days: (**a**) PC-Z; (**b**) S02b-0; (**c**) S02b-10; (**d**) S02b-10K, S02b-10 and S02b-10K are pre-absorbed, and S02b-0 is non-absorbed.

Figure 9 shows the influence of SAP on the micro morphology of concrete at 56days age. Figure 9a shows that the amount of pores on the cement matrix surface of PC-Z reference concrete are more and the diameter is relatively large. Netlike hydration products grow freely, and the number of ettringite and Ca(OH)_2_ crystal is more. The surface hydration reaction of fly ash is low and there is less attachments on the surface. The surface of concrete is not dense and the structure is relatively loose. Compared with the PC-Z reference concrete, the volume of 0.2% SAP-b concrete with different ways of entrained water is shown in Figure 9b–d. The micro structure of the surface is dense and no large amounts of Ca(OH)_2_ and ettringite enrichment are found. There is a dense product C-S-H gel of the second hydration. And the fly ash is wrapped with a large number of dense gels. The internal curing effect of SAP contributes to the second hydration reaction of fly ash as well as improving the compactness of concrete microstructure. This is because the SAP absorbs additional internal curing water before the addition to mixture or absorbs free water after the addition to mixture. And then SAP gradually releases water, which needed for the second hydration reactions within the concrete. This makes the active SiO_2_ and Al_2_O_3_ of fly ash well involved in the hydration reaction, and consumes a large amount of Ca(OH)_2_. It can contribute to the formation of dense and uniform gel, improve the compactness of concrete as well as improve the durability and mechanical properties of SAP concrete. Compared to S-10 and S-0, fly ash microspheres in S-10K are wrapped with a large amount of C-S-H gel, which is the production of second hydration reaction. It explains that fly ash can play a better role in the pozzolanic effect, and further explains the reasons for the increase of compressive strength and durability.

## 4. Conclusions

The influence of volume, particle size, and ways of entrained water of SAP on the workability, compressive strength, shrinkage, carbonation resistance and chloride penetration resistance of concrete has been studied through macroscopic and microscopic test. Based on mercury intrusion porosimetry (MIP) test, X-ray diffraction (XRD) test and scanning electron microscope (SEM) test results, the pore structure, morphology of hydration products and mineral compositions of concrete have been further analyzed, and the mechanism of SAP on the performance of concrete has been studied. The main conclusions are as follows:

1. With the incorporation of SAP, the slump of concrete is influenced by SAP volume and different entrained ways of internal curing water. The pre-absorbed SAP increase the slump of mixture. And with the increase of SAP volume, the slump increases gradually. But the SAP without water absorption and the pre-absorbed SAP with the deduction of internal curing water from mixing water can reduce the slump of mixture. And with the increase of SAP volume, the decreasing range increased.
2. At early age, the concrete mixed with SAP does not have effect on internal maintenance. Then the internal curing effect of SAP gradually begins to work. At later age, the internal curing effect of SAP is more obvious, and the compressive strength of the internal curing concrete with SAP increases continuously. In addition, SAP with the particle size of 150–180 μm has an optimum particle size range to improve the compressive strength of concrete.
3. The SAP can alleviate the total shrinkage of concrete. When the non-absorbed SAP or pre-absorbed SAP with the deduction of internal curing water from mixing water are added, the total shrinkage value of SAP concrete decreases slightly. However, the effect of reducing total shrinkage of concrete by adding pre-absorbed SAP is remarkable.
4. SAP can significantly improve the abilities of carbonation resistance and chloride penetration resistance of concrete. And different ways of entrained water have great impact on the abilities of carbonation resistance and anti-chloride erosion of concrete. When the pre-absorbed SAP with the deduction of internal curing water from mixing water is added, the abilities of carbonation resistance and anti-chloride erosion of concrete are relatively better.
5. When SAP is added into concrete, the gel pores and small capillary pores increase, the size of big capillary pores and air pores are reduced. The pre-absorbed SAP can increase the porosity and total pore volume of concrete. But the SAP without water absorption and the pre-absorbed SAP with the deduction of internal curing water from mixing water can reduce the porosity and total pore volume of concrete.

## Figures and Tables

**Table 1:** Properties of ordinary Portland cement.

Setting time/min		Flexural strength/MPa		Compressive strength/MPa	
Initial setting	Final setting	3d	28d	3d	28d
158	198	6	8.7	23.6	48.9

**Table 2:** Composition of cement and fly ash (%).

	SiO_2_	Fe_2_O_3_	Al_2_O_3_	SO_3_	MgO	CaO	Loss
Cement	19.24	3.25	4.08	4.81	4.19	62.47	2.53
Fly ash	51.81	4.69	32.91	1.65	1.36	4.697	4.19

**Table 3:** Properties of superabsorbent polymers.

SAP	Particle size (μm)	Water absorption (g/g)	Water desorption (%)
a	425–250	115	4.5
b	180–150	90	6

**Table 4:** Mix proportion of concrete (kg/m³).

Sample	Cement	Fly ash	Coarse aggregate	Sand	Water	SAP	Internal curing water	Wic/B
PC	405	45	1032	747	171	0	0	0
S01a-0	405	45	1032	747	171	0.45	0	0
S01b-0	405	45	1032	747	171	0.45	0	0
S01a-10	405	45	1032	747	171	0.45	4.5	0.01
S01b-10	405	45	1032	747	171	0.45	4.5	0.01
S01a-10K	405	45	1032	747	166.5	0.45	4.5	0.01
S01b-10K	405	45	1032	747	166.5	0.45	4.5	0.01
S02a-0	405	45	1032	747	171	0.9	0	0
S02b-0	405	45	1032	747	171	0.9	0	0
S02a-10	405	45	1032	747	171	0.9	9	0.02
S02b-10	405	45	1032	747	171	0.9	9	0.02
S02a-10K	405	45	1032	747	162	0.9	9	0.02
S02b-10K	405	45	1032	747	162	0.9	9	0.02
S03a-0	405	45	1032	747	171	1.35	0	0
S03b-0	405	45	1032	747	171	1.35	0	0
S03a-10	405	45	1032	747	171	1.35	13.5	0.03
S03b-10	405	45	1032	747	171	1.35	13.5	0.03
S03a-10K	405	45	1032	747	157.5	1.35	13.5	0.03
S03b-10K	405	45	1032	747	157.5	1.35	13.5	0.03

Note: Wic is water introduced for internal curing.

**Table 5:** Result of mercury intrusion porosimetry (MIP) analysis for cement paste at 56 days.

Samples	Total pore volume (mL/g)	Porosity (%)	Pore size distribution (mL/g)			
			3–10 nm	10–100 nm	100–1000 nm	>1000 nm
PC-Z	0.0675	12.3197	0.0087	0.0284	0.0158	0.0146
S02b-0	0.0611	11.2134	0.0102	0.0248	0.0128	0.0133
S02b-10	0.0704	12.6239	0.0119	0.0332	0.0142	0.0111
S02b-10K	0.0621	11.2504	0.0108	0.0329	0.0124	0.0060

Note: S02b-10 and S02b-10K are pre-absorbed, and S02b-0 is non-absorbed.

**Table 6:** The amount of Ca(OH)_2_ and ettringite in the cement pastes at 56 days.

Samples	Ca(OH)_2_ (%)	Ettringite (%)
PC-Z	13.6	4.48
S02b-0	7.17	2.28
S02b-10	8.05	2.55
S02b-10K	7.24	2.49

Note: S02b-10 and S02b-10K are pre-absorbed, and S02b-0 is non-absorbed.
